# A municipality-based vocational rehabilitation programme for occupationally marginalized citizens: a study protocol for a mixed methods study

**DOI:** 10.1186/s12913-018-3322-4

**Published:** 2018-07-03

**Authors:** Lotte Nygaard Andersen, Mette Jensen Stochkendahl, Kirsten Kaya Roessler

**Affiliations:** 1 Department of Sports Science and Clinical Biomechanics, University of Southern Denmark, Odense, Denmark; 2 Nordic Institute of Chiropractic and Clinical Biomechanics, Odense, Denmark; 3 Department of Psychology, University of Southern Denmark, Odense, Denmark

**Keywords:** Vulnerable citizens, Unemployed, Social worker, Interdisciplinary rehabilitation, Work ability, Work force, Longitudinal survey, Interview, Social service interventions

## Abstract

**Background:**

In 2013 vocational rehabilitation programmes (VRP) were given official and legal approval under Danish law to assist occupationally marginalized citizens in gaining general life skills, building their work ability, and increasing their chances of entering the work force. The project’s aim is to develop a detailed understanding of the health, psychosocial and work circumstances of participating citizens, and of the important processes and mechanisms underlying the potential effects of participating in the VRP.

**Methods:**

This study uses an exploratory mixed methods approach with sequential use of quantitative and qualitative methods. Participants are citizens assigned to an individually tailored VRP in the municipality of Sonderborg, Denmark. The quantitative part of the study consists of a longitudinal survey in which participants complete questionnaires at baseline and at follow-up one year later. Variables include demographic and personal characteristics, the latter ascertained through validated questionnaires on well-being, physical activity, interpersonal problems, general health, work ability, kinesiophobia, self-efficacy, depression and anxiety. The qualitative part of the study consists of semi-structured interviews and observations that explore experiences related to VRP. Participants will be recruited and data collected from questionnaires, interviews and observations in the period February 2016 – March 2018.

**Discussion:**

This research will assemble a unique corpus of knowledge about the characteristics, experiences and outcomes of occupationally marginalized citizens participating in a VRP. It will identify potential enablers and barriers to a successful outcome, and ultimately this knowledge will help inform the future design of individually tailored VRP’s.

**Trial registration:**

ClinicalTrials.gov Identifier: NCT02641704, date of registration December 29, 2015.

## Background

Vocational rehabilitation for those situated on the margins of the work force is a challenge in most Western societies. For example, although the majority wish to work [1], international studies show that as few as 15% of people with severe mental health problems hold gainful employment [2, 3]. In Sweden, almost half of participants on various vocational rehabilitation programmes have ended up with a disability pension [4]. This strongly belies the notion that labour participation is an important element in recovery, regardless of the reason for unemployment or the individual’s underlying condition, and that work is closely linked with better quality of life [5, 6].

The challenge of obtaining and retaining a job has only rarely been evaluated for various groups of marginalized citizens. For people with severe mental illness, a recent meta-analysis has shown that vocational employment, individually designed and supported, is an effective avenue to employment [7]. For example, the positive findings of several individual placement and support studies [8, 9], which consider competitive employment as the only outcome of interest, have promoted further discussions of the way vocational rehabilitation is offered. Evidence also supports the positive effects on employment of information being exchanged between stakeholders from the healthcare sector and work place [10]. Therefore, there is a need for more research that can tie interventions related to health and employment closer together [11, 12]. In addition, more research is needed on the role of rehabilitation professionals and on their willingness to collaborate with relevant key stakeholders, such as vulnerable citizens and their employers [13].

In Denmark, vocational rehabilitation programmes (VRP) are provided by the local authorities (municipalities) and set within the tax funded social service system. They are designed to assist vulnerable, unemployed, and occupationally marginalized individuals in a structured manner, the objective being to increase their chances to entering the work force. To this end the programmes providing general life and social skills and build up work ability through individually tailored, holistic interventions. Citizens eligible for VRP are defined as those struggling over prolonged periods of time, often years, with multiple and/or significant issues, including physical health problems, mental disorders and/or social issues, which render them vulnerable and marginalized. They are potential candidates for early retirement benefits, but these can only be approved, if a VRP of between one and five years has not been successful [14].

The VRP’s are therefore individually tailored programmes designed to help citizens develop their personal skills that will increase their chances of entering the work force.

The programme were officially ratified by the Danish Government in 2013 [15]. A Danish report [16] showed that 90% of participants in VRP’s had occupational experience, 95% of the participants already receive government benefits, out of which 60% receive sickness absence benefits. The report also stated that conditions related to health, both physical and mental, are very often barriers to employment, further education or re-training [16]. Despite this report, there is still significant lack of knowledge about the personal characteristics and profiles of citizens assigned to VRP, including their everyday life situations and of their mental and physical health conditions, and, ultimately, about the impact on individuals and the outcomes of the VRP.

This paper describes the design of an exploratory mixed methods study, which based on studies in the social service sector in Denmark, will contribute knowledge about occupationally marginalized citizens and their participation in VRP’s.

### Research aim and objectives

The aim of this study is to develop a detailed understanding of citizens assigned to an interdisciplinary VRP in one municipality in Denmark, covering their health, their psychosocial and work situation, and significant processes and mechanisms underlying the potential effects of participating in VRP.

The objectives are :

1. to develop a descriptive profile of the citizens assigned to a VRP in Sonderborg Municipality
2. to obtain an understanding of the potential developmental path citizens take when participating in a VRP
3. to examine how citizens experience the impact of the VRP
4. to explore the role of social interactions on the citizens’ path of development.

## Methods/design

### Study design

A mixed-method exploratory approach [17] with the combined use of quantitative and qualitative methods will be used in this study, because the research objectives are complex and comprehensive in nature and they require multiple outcomes. A range of data will need to be assembled and analysed to provide a sufficiently nuanced picture if the project is to achieve its objectives. This combined methodological approach uses a variety of data collection methods. These will be described in part A and part B below.

### Participants

Eligible participants are assigned to a VRP in Sonderborg Municipality. Criteria for acceptance on a VRP are as follows: a) Participants should be unemployed citizens aged 18 to 65 years, b) They have been assessed as being vulnerable and occupationally marginalized because of complex challenges around one or more issues, such as social problems, physical conditions or mental disorders, c) The complex nature of their problems and their situation means that they are not directly available for the labour market, candidates for continued education or retraining of skills, and d) They are at high risk of early retirement.

### Study settings

Towards the end of 2015, in accordance with Danish legislation on VRP, Sonderborg Municipality in the southern part of Denmark (population approximately 75,000) established a *Competency and Integration Center (CIC)* within the social services department. The CIC initiates and coordinates VRP’s based on an expected enrolment of approximately 400 citizens per year. The VRP’s offered by Sonderborg Municipality are individually tailored, multifaceted programmes, which are designed so they can contain different types of intervention related to employment, health and the management of everyday life.

Because the content of the VRP’s is tailored to the individual citizens, the local Health Care Centre as well as local educational institutions and companies may be involved in executing elements of VRP in collaboration with the CIC.

### Vocational rehabilitation program

#### Eligibility for inclusion in VRP

**Fig. 1:**
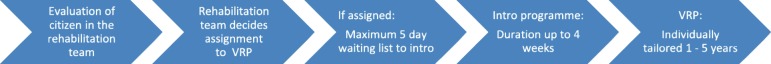
Timeline for participation in VRP in sonderborg municipality

Citizens’ eligibility for the VRP is determined by a social worker at the Job Center. In cooperation with the citizen, the social worker submits an application for the citizen to be included in a VRP programme. The application is sent to an interdisciplinary rehabilitation team in the municipality with an individual, targeted plan for rehabilitation. This plan is discussed at a meeting with the citizen, the social worker and the rehabilitation team, and the rehabilitation team may suggest alternatives. The rehabilitation team makes a final decision about granting a VRP only after consultation and dialogue with the citizen. The process is described in Fig. 1.

#### Programme content

Each citizen in the programme is assigned a coordinative social worker. Throughout the course of the programme, the social worker assists the citizen in tailoring and carrying out the programme. The programmes are individually tailored, involve a holistic view of the citizen’s situation, are interdisciplinary and aim to build up the citizens’ work ability. The following are examples of the activities that can be included in the programmes, either as a single component or in combination:

- Training in social skills (e.g. Leisure activities, company internship programs, voluntary work)
- Municipality based support services (e.g. Providing a supporting contact person and/or mentor)
- Courses in self-management or in supervision of issues related to mental or physical health (e.g. Chronic pain, anxiety, depression and post-traumatic stress disorder, physical activity, overweight, dietary habits or smoking)
- Treatment for alcohol and drug abuse
- Rehabilitation after chronic disease (e.g. Cancer, chronic obstructive lung disease, arthritis, musculoskeletal disorders or heart diseases)
- Employment integration services (e.g. Internship programmes)
- Educational services (e.g. Supervision, school-leaving examination and supplementary education)
- Family therapy services

### Methods part A

This part is a longitudinal survey using postal questionnaires at baseline and at one-year follow-up.

### Inclusion of participants and time sequences

Participants will be drawn from databases containing all citizens assigned to VRP in Sonderborg Municipality. At the start of the project period, data on citizens assigned to a VRP during the previous year will be retrieved. After that, we will continue to retrieve lists with citizens assigned to the programme.

**Fig. 2:**
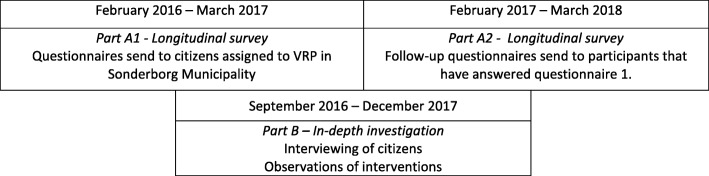
Timeline for data collection

In Part A, citizens will be recruited to fill out questionnaires from February 2016 to March 2017. Follow-up questionnaires will be sent February 2017 – March 2018. A timeline for data collection is presented in Fig. 2.

### Data collection methods for part A – Quantitative

Questionnaires will be sent via postal mail to all citizens assigned to VRP at baseline (A1) and at follow-up (A2) after one year. The questionnaire is designed to add to the descriptive profile of the participants as regards their gender, age, height, body weight and demographic characteristics, such as educational qualifications and domestic situation.

#### The physical domain

*Work ability* will be measured using a single question from the Work Ability Index [18] “Imagine that your work ability is worth 10 points when it is at its best. How many points would you give your present work ability?”. The answer will be recorded on an 11-point numeric rating scale from zero to ten, where zero represents ‘not able to work’ and ten ‘highest work ability’ [19, 20]. This single question has previously been shown to adequately assess the status and progress of work ability [20].

*General health* will be measured using two selected items from the SF-36 Health Survey concerning self-perceived general health (at present and one year ago) “In general, would you say your health is” and “Compared to one year ago, how would you rate your health in general now?” Answers will be recorded using a 5-point Likert Scale ranging from excellent to poor and from much better now than one year ago to much worse than one year ago [21, 22].

Five components of *physical fitness* will be evaluated using a numeric rating scale with illustrations and verbal anchors for the extremes. There is one question and it relates to each of the five components: aerobic fitness, muscle strength, endurance, flexibility and balance. “How would you rate the following components of physical fitness compared with others of your own age and sex?”. Answers will be recorded on an 11-point numeric scale ranging from weak to strong or poor to good [23, 24]. The questions have been translated into Danish and validated [23].

*Physical activity* will be measured with a single question “This question is about how much you move and exert yourself physically during your leisure time?” from the Saltin-Grimby Physical Activity Level Scale. The answer is recorded on a 4-point Likert scale measuring a range from almost completely passive to strenuously active [25–27].

*Pain* will be measured on a Visual Analogue Scale (VAS) using a 100 mm VAS anchored with ‘no pain’ at 0 mm and ‘worst imaginable pain’ at 100 mm during the last 7 days [28].

#### The psychological domain

*Well-being* will be measured using the WHO-5 Well-Being Index [29, 30] which is a questionnaire that measures current mental well-being. It is a 5-item questionnaire originally developed from the 28-item WHO Well-Being Questionnaire and it has been shown to have both clinical and psychometric validity [31].

*Depression and anxiety* will be measured using the Patient Health Questionnaire-4 for Depression and Anxiety (PHQ-4). It is an ultra-brief screening instrument consisting of four items, which includes two validated two-item screeners (PHQ-2 and GAD-2). Answers will be measured on a 4-point Likert scale measuring a range from ‘not at all’ to ‘nearly every day’ [32, 33].

*Loneliness* will be measured using the *T-ILS* [34] for loneliness. It is a short version of the longer UCLA version and it consists of three items which have shown adequately agreement with the UCLA version to assess loneliness [35, 36]*.* Answers will be measured on a 3-point Likert scale measuring the range ‘Hardly ever’, ‘Some of the time’ and ‘often’.

*Self-efficacy* will be measured using the 10-item psychometric scale ‘The General Self-Efficacy Scale’ [37] which is designed to assess the belief in one’s competence to cope with a broad range of stressful or difficult demands in life [38]. Answers will be measured at a 4-point Likert Scale ranging from ‘not at all true’ to ‘exactly true’ [29, 37, 39].

*Kinesiophobia* refers to dysfunctional beliefs about physical activity, and it has been conceptualized as a fear of movement or (re)injury by Vlaeyen et al. [40]. Pain-related fear has previously been shown to be a valid predictor of chronic pain and disability [41]. Kinesiophobia will be measured using the Tampa Scale for Kinesiophobia [42–44]. The scale consists of 17 items each of which can be answered on a 4-point Likert Scale ranging from strongly agree to strongly disagree [45].

#### The social domain

*Interpersonal problems* will be investigated using ‘The Inventory of Interpersonal Problems’ [46, 47], which is a questionnaire consisting of 64 items that identify a subject’s most salient interpersonal difficulties. Answers will be recorded on eight scales and scored on 5-point Likert Scales. These scales range between areas that may be difficult for a subject to engage in to areas where the subject may engage too much.

### Study size

Over the duration of the project, we expect to include 150 participants. Due to its exploratory character, we will not consider issues of statistical power in this study.

### Methods part B

#### Inclusion of participants and time sequences

Participants in Part B will be drawn from the pool of participants in Part A, recruited through personal contact during observations, or, if required, municipality employees will be asked to identify relevant informants for interviews. The situations to be observed will be selected in cooperation with employees in the CIC in Sonderborg Municipality.

The approach that will be used involves purposeful selection covering variation between informants in interviews and in situations for observations [48, 49]. During the study period, we will keep a continuous eye on the research objectives to ensure that selected informants and situations are relevant and information-rich [49]. The selection strategy will enable us to identify similarities and differences in data and develop depth in the understanding of the objectives. However, at the present stage of the study the variations among the citizens are unknown, and that means that an iterative selection will be used to capture variation.

In part B of the study we will recruit participants for interviewing and conduct observations during the period September 2016 – December 2017. The time sequences are illustrated in Fig. 1.

### Data collection methods for part B - qualitative

Initially, exploratory, individual, semi-structured interviews will be conducted. Interviews will be conducted to grasp the essence of being a VRP participant. We will follow coordinative social workers in their interaction with VRP participants and carry out observations of selected programme components. The purpose of using multiple qualitative data collection methods is to gain information about different aspects of the processes involved in citizens participation in VRP [48].

#### Semi-structured interviews

The interview guide will be prepared on the basis of our research objectives and include questions enabling us to produce a descriptive profile of the participants and develop an understanding about the processes involved in their participation in the programme. As part of the preparation for the interview guide we will also review the scientific literature about patient-centeredness [50], recovery [51, 52], rehabilitation [53], and Danish web resources about VRP [14]. The interview questions will be formulated in the interview guide [48], but the guide itself will continue to evolve throughout the study period as new insights are gained and new questions emerge. During interview we will be meticulous in asking participants about specific events and actions directly related to VRP rather than questions that only require answers in the form of generalizations or abstract opinions [48]. Throughout, we will steer and balance the interview to ensure that it is centred on issues related to the specific research objectives while at the same time giving the participant opportunities to bring up relevant and significant issues that were not anticipated. Interviews will be digitally audio recorded, transcribed verbatim and reviewed twice to check transcription accuracy.

*Observations* of meetings between citizens and coordinators of VRP, and observations of components in the VRP will be used as complements to interviews. The observations will be used to open up the qualitative study [48], make descriptions of social worker practice (settings, behaviour, events and interactions), and give ideas and insider knowledge. The knowledge acquired from the observations also will translate into relevant context specific questions for the interviews. During observations we are interested in how actions and interactions constitute relationships, and how these influences the citizens’ personal development. During observation sessions, a note-taking strategy will be used in the natural scene, aiming to be minimally intrusive. After each observation, the results will be evaluated and form the basis for reflections on the next step towards gaining relevant information related to research objectives.

### Study size

In part B, we expect to do around 10 h of observations, 10–15 semi-structured interviews with participants, and 2–5 interviews with social workers. To guide the study size in this part the interviews will be reviewed continuously during the study process to appraise information power. Preliminary analysis of interviews will support the sampling procedure. The number of interview participants will be adjusted according to information power related to our study objectives aiming at understanding the processes operating when participating in VRP [48, 54].

### Data analysis – Part A and B

During the data analysis, the different types of data will be integrated at different stages. First, quantitative data will be presented pictorially (e.g. graphs and tables). After data display, a data transformation stage will follow where results from the quantitative part of the study will be transformed into a summary that can be merged with the qualitative findings containing participants’ descriptions of their situations and participation in VRP. The various data types will be compared for similarities and contrasts allowing us to explore, discover and develop knowledge about participants and their participation in VRP and about the relationships between the persons involved. Finally, data will be integrated, interpreted and gathered together into a coherent whole for accomplishing the aim of this study [55].

#### Data from questionnaires

Baseline characteristics will be reported as percentages for binary and categorical variables and as mean values and standard deviation for continuous variables. An evaluation with regression techniques, generalized estimating equations and path analysis will be used to explore associations between measured variables at different time points. Statistical significance will be accepted at *p*-values < 0.05.

#### Data from interviews and observations

The main strategy for analysis of the transcripts from the interviews and observations will be as outlined here. The steps are: 1) Transcription of observation and interview material, 2) Careful reading of interview transcripts and observational notes giving a total impression of statements in the material, 3) Coding as the main categorizing strategy. We will look for similarities and differences in the descriptions provided by the interviewees, both between individuals and across interviews. This inductive categorical sorting of the material will lend weight to the emerging themes, 4) Condensation – from code to meaning, summarizing each while maintaining constant focus on the research objectives, 5) Synthesizing and developing descriptions and concepts [48, 56]. These analytical steps will ensure methodological rigour and transparency both in the analytical process and in the reporting of results. Quotations from citizens or municipal employees will be used to support claims and illustrate points.

### Ethical considerations

This study is declared exempt from approval by the Regional Scientific Ethics Committee for Southern Denmark. In the qualitative part of the study, all participant information and data collected from the municipality and the questionnaires will be handled according to the approval by The Danish Data Protection Agency. Questionnaires will be anonymized and stored in a locked filing cabinet in a locked room at the University of Southern Denmark. All data will be fed in and stored in a secure, password protected drive on a university server. Only the research team will have access to the data. In the qualitative part of the study, informed consent regarding participation in the interview will be obtained from all participants on the day of the interview. Confidentiality and anonymity will be maintained through the use of identification codes.

## Discussion

To our knowledge this will be one of the first research projects to study citizens’ participation in VRP in Denmark. The results of the study will provide detailed characteristics of citizens on a VRP and deepen our understanding of the complex situations that many of these citizens find themselves in. The study will help us acquire insight into the participants’ experience of their VRP, and their perceptions of the programme components and personal interactions with social workers and other stakeholders. Finally, we will be able to evaluate any change in work ability and potential influence of the VRP on the participants’ work-life situation.

Importantly, this study will not only provide knowledge about the citizens’ situations and the current use of the VRP, but will also be a necessary step in the generation of new ideas for the planning of future VRP’s. The information will have the potential to inform citizens, municipalities, occupational stakeholders, the healthcare sector, politicians and other decision-makers about the design and implementation of interventions for occupationally marginalized citizens.

We have chosen a mixed methods approach that uses a complementary combination of quantitative and qualitative data collection methods in an embedded design. This combination of methods can provide both breadth in the description of the citizens and depth in the investigation and understanding of experiences for the citizens that participate in the VRP’s. This combination can provide much more complete data about citizens and their participation in the municipality-based programme than a single-stranded methodological approach.

The project will be conducted in Sonderborg Municipality. However, it is expected that the conclusions from this study will be transferable to similar contexts and settings, e.g. other geographical municipality settings, because of the relatively small cultural diversity in Denmark. The concept of validity will be handled in different ways during the study period and different types of validity issues will be relevant to address. For example, one type of validity for the qualitative part will be ensured by preparation, i.e. non-leading questions will be prepared and used during interviewing. During interviewing and subsequent analysis, we will be loyal to informants and avoid over-interpretations. Only validated questionnaires will be used in the quantitative part to describe the citizens’ situation. To ensure quality and rigour in the analysis and transparency in reporting the study, we will continually review our methods according to generally approved guidelines and check-lists for observational, qualitative and mixed methods research [55, 57, 58].

## Abbreviations

CIC: Competency and Integration Center
VRP: Vocational Rehabilitation Programme

## References

[1] Bond GR, Drake RE (2014). Making the case for IPS supported employment. Admin Pol Ment Health.

[2] Harnois G, Gabriel P. Mental health and work: impact, issues and good practices: World Health Organization; 2000.

[3] Marwaha S, Johnson S, Bebbington P, Stafford M, Angermeyer MC, Brugha T (2007). Rates and correlates of employment in people with schizophrenia in the UK, France and Germany. Br J Psychiatry.

[4] Ahlgren A, Broman L, Bergroth A, Ekholm J (2005). Disability pension despite vocational rehabilitation? A study from six social insurance offices of a county. International journal of rehabilitation research Internationale Zeitschrift fur Rehabilitationsforschung Revue internationale de recherches de readaptation.

[5] McGurk SR, Mueser KT, DeRosa TJ, Wolfe R (2009). Work, recovery, and comorbidity in schizophrenia: a randomized controlled trial of cognitive remediation. Schizophr Bull.

[6] Sickness and Disability Schemes in the Netherlands. Country memo as a background paper for the OECD Disability Review. 2007. In*.* Paris: OECD; 2007.

[7] Modini M, Tan L, Brinchmann B, Wang MJ, Killackey E, Glozier N (2016). Supported employment for people with severe mental illness: systematic review and meta-analysis of the international evidence. Br J Psychiatry.

[8] Bond GR, Drake RE, Becker DR (2012). Generalizability of the individual placement and support (IPS) model of supported employment outside the US. World Psychiatry.

[9] Bond GR, Drake RE, Campbell K (2016). Effectiveness of individual placement and support supported employment for young adults. Early intervention in psychiatry.

[10] Høgelund J (2012). Effekter af den beskæftigelsesrettede indsats for sygemeldte. En litteraturoversigt. In.

[11] Brussig M, Dragano N, Mumken S (2014). Health promotion for unemployed jobseekers: new developments in Germany. Health Policy.

[12] Kellett S, Bickerstaffe D, Purdie F, Dyke A, Filer S, Lomax V (2011). The clinical and occupational effectiveness of condition management for incapacity benefit recipients. Br J Clin Psychol.

[13] Wltavsky Z, Lebar L, Bitenc C (2014). Evaluation of competencies in the field of vocational rehabilitation and the employment of persons with disabilities. International journal of rehabilitation research Internationale Zeitschrift fur Rehabilitationsforschung Revue internationale de recherches de readaptation.

[14] Hvad er rehabiliteringsforløb https://www.borger.dk/arbejde-dagpenge-ferie/Fleksjob-loentilskud-for-foertidspensionister-revalidering/ressourceforloebsydelse-under-ressourceforloeb. Accessed 27 June 2018.

[15] LOV nr 1380. Lov om ændring af lov om en aktiv beskæftigelsesindsats, lov om aktiv socialpolitik, lov om social pension og forskellige andre love (Reform af førtidspension og fleksjob, herunder indførelse af ressourceforløb, rehabiliteringsteam, fleksløntilskud m.v.) https://www.retsinformation.dk/forms/r0710.aspx?id=144932. Accessed 27 June 2018.

[16] Status på kommunernes implementering af førtidspensions- og fleksjobreform http://www.cabiweb.dk/media/652237/evaluerings-af-kommunernes-implementering-af-foertidspensions-og-fleksjobordningen.pdf. Accessed 27 June 2018.

[17] Creswell JW, Plano Clark VL. Designing and conducting mixed methods research. In: SAGE Publications. 2014;

[18] Ilmarinen J. The work ability index (WAI). Occup Med. 2007;57(2):160–0.

[19] Ilmarinen J (2009). Work ability - a comprehensive concept for occupational health research and prevention. Scand Journal of Work Environment & Health.

[20] Ahlstrom L, Grimby-Ekman A, Hagberg M, Dellve L (2010). The work ability index and single-item question: associations with sick leave, symptoms, and health - a prospective study of women on long-term sick leave. Scand Journal of Work Environment & Health.

[21] Bjorner JB, Thunedborg K, Kristensen TS, Modvig J, Bech P (1998). The Danish SF-36 health survey: translation and preliminary validity studies. J Clin Epidemiol.

[22] Bjorner JB, Damsgaard MT, Watt T, Groenvold M (1998). Tests of data quality, scaling assumptions, and reliability of the Danish SF-36. J Clin Epidemiol.

[23] Stroyer J, Essendrop M, Jensen LD, Warming S, Avlund K, Schibye B (2007). Validity and reliability of self-assessed physical fitness using visual analogue scales. Percept Mot Skills.

[24] Rasmussen CDNM, Andersen LLP, Clausen TP, Stroyer JP, Jorgensen MBP, Holtermann AP. Physical capacity and risk for long-term sickness absence: a prospective cohort study among 8664 female health care workers. Journal of Occupational & Environmental Medicine. 10.1097/JOM.0000000000000395 25749130

[25] Saltin B, Grimby G. Physiological analysis af middle-aged and old former athletes. Circulation. 1968;38 10.1161/01.cir.38.6.1104 5721960

[26] Rodjer L, Jonsdottir IH, Rosengren A, Bjorck L, Grimby G, Thelle DS (2012). Self-reported leisure time physical activity: a useful assessment tool in everyday health care. BMC Public Health.

[27] Grimby G, Borjesson M, Jonsdottir IH, Schnohr P, Thelle DS, Saltin B (2015). The "Saltin-Grimby physical activity level scale" and its application to health research. Scand J Med Sci Sports.

[28] Huskisson E (1974). Measurement of pain. Lancet.

[29] Christensen TN, Hansen ML. Validerede instrumenter ved måling af mental sundhed - til evalueringer på det sociale område. Socialstyrelsen. 2014;

[30] Guide til trivselsindekset: WHO-5 https://sundhedsstyrelsen.dk/~/media/874C7A337C5F4450B55476CA535461E3.ashx. Accessed 27 June 2018.

[31] Bech P, Olsen LR, Kjoller M, Rasmussen NK (2003). Measuring well-being rather than the absence of distress symptoms: a comparison of the SF-36 mental health subscale and the WHO-five well-being scale. Int J Methods Psychiatr Res.

[32] Kroenke K, Spitzer RL, Williams JB, Lowe B (2009). An ultra-brief screening scale for anxiety and depression: the PHQ-4. Psychosomatics.

[33] Lowe B, Wahl I, Rose M, Spitzer C, Glaesmer H, Wingenfeld K (2010). A 4-item measure of depression and anxiety: validation and standardization of the patient health Questionnaire-4 (PHQ-4) in the general population. J Affect Disord.

[34] Hughes ME, Waite LJ, Hawkley LC, Cacioppo JT (2004). A short scale for measuring loneliness in large surveys: results from two population-based studies. Research on aging.

[35] Russell DW (1996). UCLA loneliness scale (version 3): reliability, validity, and factor structure. J Pers Assess.

[36] Lasgaard M (2007). Reliability and validity of the Danish version of the UCLA loneliness scale. Personal Individ Differ.

[37] General Self-Efficacy Scale http://userpage.fu-berlin.de/~health/selfscal.htm#Top%20of%20Page. Accessed 27 June 2018.

[38] Luszczynska A, Scholz U, Schwarzer R (2005). The general self-efficacy scale: multicultural validation studies. The Journal of psychology.

[39] Schwarzer R, Jerusalem M (1995). Generalized self-efficacy scale. Measures in health psychology: A user’s portfolio Causal and control beliefs.

[40] Vlaeyen JWS, Kole-Snijders AMJ, Rotteweel AM, Ruesink R, Heuts PHTG (1995). The role of fear of movement/(re)injury in pain disability. J Occup Rehabil.

[41] Leeuw M, Goossens ME, Linton SJ, Crombez G, Boersma K, Vlaeyen JW (2007). The fear-avoidance model of musculoskeletal pain: current state of scientific evidence. J Behav Med.

[42] Kori S, Miller R, Todd D (1990). Kinesiophobia: a new view of chronic pain behavior. Pain management.

[43] Jorgensen MB, Damsgard E, Holtermann A, Anke A, Sogaard K, Roe C (2015). Properties of the Tampa scale for Kinesiophobia across workers with different pain experiences and cultural backgrounds: a Rasch analysis. Journal of applied measurement.

[44] Vlaeyen JW, Kole-Snijders AM, Boeren RG, van Eek H (1995). Fear of movement/(re)injury in chronic low back pain and its relation to behavioral performance. Pain.

[45] Lundberg MKE, Styf J (2003). Carlsson SG. A psychometric evaluation of the Tampa scale for Kinesiophobia - from a physiotherapeutic perspective. Physiotherapy theory and practice.

[46] Horowitz LM, Rosenberg SE, Baer BA, Ureno G, Villasenor VS (1988). Inventory of interpersonal problems: psychometric properties and clinical applications. J Consult Clin Psychol.

[47] Alden LE, Wiggins JS, Pincus AL (1990). Construction of circumplex scales for the inventory of interpersonal problems. J Pers Assess.

[48] Maxwell JA. Qualitative research design: an interactive approach, vol. 41: Sage; 2013.

[49] Palinkas LA, Horwitz SM, Green CA, Wisdom JP, Duan N, Hoagwood K (2015). Purposeful sampling for qualitative data collection and analysis in mixed method implementation research. Admin Pol Ment Health.

[50] Scholl I, Zill JM, Harter M, Dirmaier J (2014). How do health services researchers understand the concept of patient-centeredness? Results from an expert survey. Patient Preference and Adherence.

[51] Le Boutillier C, Leamy M, Bird VJ, Davidson L, Williams J, Slade M. What does recovery mean in practice? A qualitative analysis of international recovery-oriented practice guidance. Psychiatric services (Washington, DC) 2011;62(12):1470–1476. 10.1176/appi.ps.001312011 22193795

[52] Leamy M, Bird V, Le Boutillier C, Williams J, Slade M (2011). Conceptual framework for personal recovery in mental health: systematic review and narrative synthesis. Br J Psychiatry.

[53] Jesus TS, Bright F, Kayes N, Cott CA (2016). Person-centred rehabilitation: what exactly does it mean? Protocol for a scoping review with thematic analysis towards framing the concept and practice of person-centred rehabilitation. BMJ Open.

[54] Malterud K, Siersma VD, Guassora AD (2016). Sample size in qualitative interview studies: guided by information power. Qual Health Res.

[55] Leech NL, Onwuegbuzie AJ (2010). Guidelines for conducting and reporting mixed research in the field of counseling and beyond. Journal of Counseling and Development: JCD.

[56] Malterud K (2012). Systematic text condensation: a strategy for qualitative analysis. Scandinavian Journal of Public Health.

[57] Qualitative research review guidelines - RATS modified from How to peer review a qualitative manuscript. https://bmjopen.bmj.com/content/suppl/2012/01/12/bmjopen-2011-000138.DC1/BMJ_Open_IMG_Physician_Migration_RATS_Checklist.pdf. Accessed 27 June 2018.

[58] von Elm E, Altman DG, Egger M, Pocock SJ, Gøtzsche PC, Vandenbroucke JP (2007). The strengthening the reporting of observational studies in epidemiology (STROBE) statement: guidelines for reporting observational studies. Ann Intern Med.

